# Persistent Cloaca associated with Unilateral Lung Agenesis- A Rare Presentation

**Published:** 2014-04-01

**Authors:** Amit Mohanrao Uttarwar, Rakesh S Joshi, Jaishri Ramji, Mitesh Bachani, DS Rathore

**Affiliations:** B. J. Medical Collage and Civil Hospital Ahmedabad, India

**Keywords:** Persistent cloaca, Lung agenesis

## Abstract

We report a case of full term female child having persistent cloaca who was diagnosed to have right lung agenesis on investigations.

## INTRODUCTION

Persistent cloaca comprises of about 10% of all cases of anorectal malformation. More than 80% of all patients have associated urological anomalies. Patients with long channel cloaca have a high frequency of complex associated anomalies. The most common urological anomalies are renal agenesis, renal dysplasia, vesico-ureteral reflux and megaureter. About 40% of the patients have tethered spinal cord. Other associated anomalies are cardiac malformation, vertebral deformities, esophageal atresia, and gastrointestinal duplications, pouch colon, absence of uterus, lower limb deformity.[1] The association of lung aplasia/ hypoplasia is very rare and exact incidence is not known.[2]

## CASE REPORT

A 3-day old female neonate born at full term through normal vaginal delivery presented with had absent anal opening; she was passing stool from vestibule. The antenatal period was uneventful, but there was no record of ANC or antenatal ultrasonography. Local examination revealed presence of persistent cloaca. There was no palpable abdominal lump. Her respiratory rate was normal and she was maintaining adequate saturation on air. On auscultation, breath sounds were absent on right side. The chest x-ray reveled opacification of the right hemi thorax with mediastinal shift. In view of her stable cardio- respiratory status, we decided to first do a transverse colostomy for fecal diversion before further investigation for her lung condition. The perioperative course was uneventful. Postoperatively the baby was investigated for right hemithorax opacification. She underwent 2D- Echocardiogram which showed absent right pulmonary artery, tiny PDA, small ASD. Patient was discharged on 5th postoperative day on oral feeds with healthy functioning stoma. She was put on distal loop washouts and observation. Her cardio-respiratory status continued to be stable. On follow-up at 6 months age, CT angiography chest was done which was suggestive of agenesis of right pulmonary artery and pulmonary vein, right pulmonary agenesis and aberrant right subclavian artery (Fig. 1). We did a diagnostic bronchoscopy that revealed complete absence of right main bronchus and trachea continued as a single left bronchus with normal distal branching confirming the diagnosis of right lung agenesis. Since patient is asymptomatic, no intervention has been done for her lung condition. She is now waiting for definitive surgery for cloaca.

**Figure:**
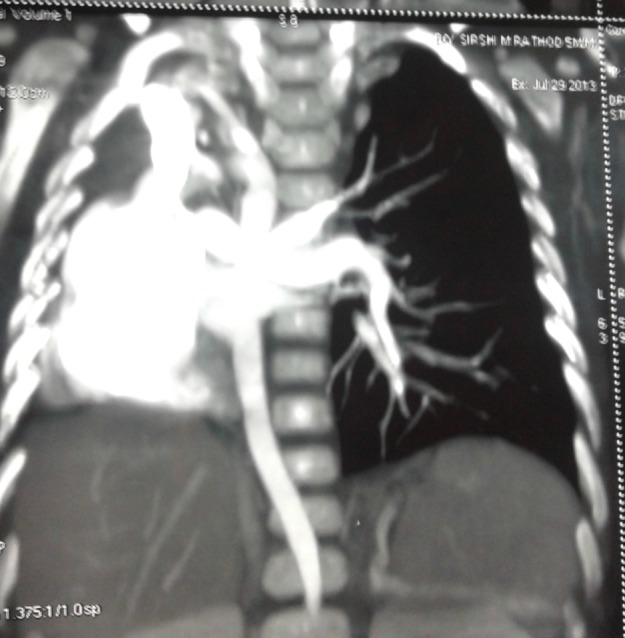
Figure 1: CT angiogram

## DISCUSSION

The incidence of unilateral pulmonary agenesis is one in every 10,000 to 15,000 autopsies. In 1762, Morgagni was first to describe the congenital underdevelopment or maldevelopment of the lungs. Since then several cases have been reported in literature. This anomaly is believed to be caused by failure of the developmental balance between the two lung buds at approximately 4th week of gestation due to unknown etiology. Schneider classified Congenital underdevelopment of the lungs as (1) Agenesis: the complete absence of the carina and the main bronchus, the lung, and the pulmonary vasculature; (2) Aplasia: the carina and the rudimentary bronchus are present, the pulmonary vessels and the alveolar tissue are absent; and (3) Hypoplasia: an ill-defined bronchus is capped by underdeveloped alveolar tissue. Nearly 50% patients have associated anomalies of other systems like vertebral, cardiac, urological, diaphragmatic defects, congenital lip anomalies and limb anomalies.[2-7]

The age of presentation is variable and depends upon extent of anomaly. Pulmonary agenesis is associated with multiple anomalies and so they often present early in neonatal period. Some patients are diagnosed when being investigated for recurrent respiratory infections; while in the majority of patients who are asymptomatic, it is an incidental finding. Diagnosis of pulmonary agenesis has been made on chest skiagram, bronchoscopy, and angiography. No treatment is required in asymptomatic cases. Treatment is necessary for chest infections. Patients having stumps (hypoplastic bud) may require surgical removal if postural drainage and antibiotics fail to resolve the infection. Corrective surgery of associated congenital anomalies, wherever feasible, may be undertaken.[2-7] Overall prognosis depends on two factors the severity of associated congenital anomalies and involvement of the normal lung in any disease process.

## Footnotes

**Source of Support:** Nil

**Conflict of Interest:** None

